# Improving lesion detection in mammograms by leveraging a Cycle-GAN-based lesion remover

**DOI:** 10.1186/s13058-024-01777-x

**Published:** 2024-02-01

**Authors:** Juhun Lee, Robert M. Nishikawa

**Affiliations:** 1 Department of Radiology, The University of Pittsburgh, 200 Lothrop Street, Pittsburgh, PA 15237 USA; 2 Department of Bioengineering, The University of Pittsburgh, 302 Benedum Hall, Pittsburgh, PA 15237 USA

**Keywords:** Lesion highlight, Convolutional neural network, Cycle generative adversarial network, Computer-aided detection

## Abstract

**Background:**

The wide heterogeneity in the appearance of breast lesions and normal breast structures can confuse computerized detection algorithms. Our purpose was therefore to develop a *Lesion Highlighter (LH)* that can improve the performance of computer-aided detection algorithms for detecting breast cancer on screening mammograms.

**Methods:**

We hypothesized that a Cycle-GAN based *Lesion Remover (LR)* could act as an *LH*, which can improve the performance of lesion detection algorithms. We used 10,310 screening mammograms from 4,832 women that included 4,942 recalled lesions (BI-RADS 0) and 5,368 normal results (BI-RADS 1). We divided the dataset into Train:Validate:Test folds with the ratios of 0.64:0.16:0.2. We segmented image patches (400 × 400 pixels) from either lesions marked by MQSA radiologists or normal tissue in mammograms. We trained a Cycle-GAN to develop two GANs, where each GAN transferred the style of one image to another. We refer to the GAN transferring the style of a lesion to normal breast tissue as the *LR.* We then highlighted the lesion by color-fusing the mammogram after applying the *LR* to its original. Using ResNet18, DenseNet201, EfficientNetV2, and Vision Transformer as backbone architectures, we trained three deep networks for each architecture, one trained on lesion highlighted mammograms (Highlighted), another trained on the original mammograms (Baseline), and Highlighted and Baseline combined (Combined). We conducted ROC analysis for the three versions of each deep network on the test set.

**Results:**

The Combined version of all networks achieved AUCs ranging from 0.963 to 0.974 for identifying the image with a recalled lesion from a normal breast tissue image, which was statistically improved (p-value < 0.001) over their Baseline versions with AUCs that ranged from 0.914 to 0.967.

**Conclusions:**

Our results showed that a Cycle-GAN based *LR* is effective for enhancing lesion conspicuity and this can improve the performance of a detection algorithm.

## Background

Breast lesions show a wide variation in size and shape and mammographically normal breast structure shows wide heterogeneity between women and often within the breast. This makes some lesions appear obvious on a mammogram, while others are subtle and difficult to detect for radiologists or detection algorithms. Specifically, breast lesions can be hidden by normal dense breast tissue and such breast cancers are often missed by radiologists and algorithms. If one can highlight such subtle lesions, it can improve the performance of lesion detection algorithms. On the other hand, global image highlights may cause some normal breast tissue to appear as a possible breast lesion, increasing the false detection rate. If we can highlight the appearance of a breast lesion while keeping a normal breast tissue as it is, we can reduce unwanted false positive detections.

In this study, we propose to use a Cycle Generative Adversarial Network (Cycle-GAN) [1] to develop a lesion highlighter. Zhu et al. [1] introduced Cycle-GAN to solve image-to-image translation (I2I) problems. I2I is about transferring images from a source domain to a target domain, while preserving the contents of the given images. I2I typically needs paired images, one from a source domain and another from a target domain. However, preparing paired images in both domains can be difficult, especially in the medical imaging field, as finding patients before and after a positive condition (e.g., cancer) is extremely difficult.

Unlike other algorithms for solving I2I (e.g., [2–4]), Cycle-GAN does not need paired datasets. If one has a mapping function of G:X → Y, such that G(X) and Y have similar characteristics, we can then define an inverse mapping F:Y → X, and a cycle consistency loss to keep F(G(X)) similar to X. Using this framework, Zhu et al. showed that Cycle-GAN can learn characteristics of two domains from unpaired image datasets and transfer the style of one domain to another and vice versa.

With properly curated image datasets from two different but related domains, we can train a Cycle-GAN to transfer the style of one image domain to another. Specifically, if we prepare image datasets of normal breast tissue and those of breast lesions from mammograms, then we can teach a Cycle-GAN to be a breast lesion remover, i.e., making an image with a breast lesion appear as an image with only normal breast tissue. If we then contrast the lesion removed image with its original, we can highlight that lesion. Through this action, we can use the lesion remover as a lesion highlighter. Hence, we hypothesized that a Cycle-GAN based lesion remover can be used as a lesion highlighter, which can improve the performance of computer-aided detection (CADe) algorithms in screening mammograms.

Since its first appearance, there have been numerous implementations and applications of Cycle-GAN on natural scene data (e.g., image to paint in various styles [1], human to robot [5], and even de-noising OCR images [6]). In the field of radiology, researchers have actively adopted Cycle-GAN to solve various tasks (diagnosis [7, 8] and segmentation [9]) for different image types (e.g., MRI [9, 10], chest X-ray [7], and mammography [8]).

Cohen et al. [10] showed that Cycle-GAN can add and remove a tumor in brain MRI images. Using the BRATS2013 synthetic MRI dataset [11, 12], they investigated how sampling bias in positive (with tumor) and negative (normal) data for training a Cycle-GAN could cause artifacts or hallucinations in GAN generated images. They prepared 1,700 MRI slices (50% with a brain tumor and another 50% without) for training and testing a Cycle-GAN for I2I between two domains, Flair and T1 weighted images. They found that the trained Cycle-GAN created unwanted artifacts (created a new tumor or removed existing tumors) in the resulting images, when there was a heavy sampling bias in the dataset, i.e., images with a specific condition (e.g., brain tumor) were dominant (90% or higher) in only one image domain.

Zhou et al. [8] studied the adversarial attack of computer-aided diagnosis (CADx) artificial intelligence (AI) algorithms in breast mammograms; how intentionally modifying the malignancy of breast lesions in mammograms (benign to malignant and vice versa) could fool a CADx-AI. They first trained a VGG11 network [13] as their example CADx-AI with an area under the ROC curve (AUC) of 0.82 using a dataset of screening mammograms with biopsy proven benign and malignant lesions from 1,284 women (918 women with benign lesions and 366 women with malignant lesions). They then built a Cycle-GAN to adversarially change the appearance of malignant lesions to benign lesions or vice versa. They found that the Cycle-GAN modified images easily fooled their CADx algorithm, resulting in an approximately 70% incorrect diagnoses on previously correct diagnoses by the same algorithm.

Note that the above two previous studies investigated the adversarial, unwanted, and unexpected effects of a Cycle-GAN on medical image analysis. Specifically, Cohen et al. considered removing existing tumors or adding a new tumor as artifacts or hallucinations that one should avoid, especially when medical professionals (e.g., radiologists) read the resulting images for assessing a medical condition. Zhou et al. warned the community about the vulnerability of CADx-AI from adversarial or unwanted attacks by a Cycle-GAN, which should be prevented and avoided by carefully inspecting the images used for training and testing them for the tasks of interest.

However, with proper curation of datasets and choice of tasks, Cycle-GAN can allow researchers to develop simulation tools that create and remove specific medical conditions, which could potentially improve many automated computer-aided algorithms in medicine. Specifically, we can develop lesion simulators and lesion removers by training a Cycle-GAN on two image datasets in two domains, one with a lesion and another with normal tissue. For example, a lesion simulator could be used as an augmentation tool for improving the performance of CADx and Computer-aided detection (CADe) algorithms for given tasks. In this study, we focused on the usage of a lesion remover for improving the detection performance of CADe algorithms in mammograms.

## Methods

### Dataset

Under an approved IRB protocol, we collected 10,310 screening Full Field Digital Mammograms (FFDMs) from 4,832 women who visited the University of Pittsburgh Medical Center (UPMC) for routine breast cancer screening. The Selenia Dimension system (Hologic Inc, Marlborough, MA, USA) was used for all mammogram exams. We used four standard views including left–right Cranio-Caudal (CC) and left–right Medio-obilque (MLO) for this study. The dataset included 4,942 mammograms that showed a recalled lesion (BI-RADS 0) from 2,416 women and 5,368 mammograms randomly selected from exams with normal readings (BI-RADS 1) from 2,416 women. MQSA radiologists marked the location of the lesion for the recalled cases. Note that we had the BI-RADS classification information at the time of the screening only. As a result, further details about the lesions, such as pathology (benign, malignant) and types (masses, calcifications), were not available at the time of the data acquisition.

To develop the lesion remover and test its potential as a lesion highlighther for improving the performance of the lesion detection algorithm, we divided our dataset into the development and independent testing, where the development set include 3,959 mammograms of 1,909 women with recalled lesions and 4,263 mammograms of 1,429 women with normal/healthy breasts, while the testset include 983 mammograms of 507 women with recalled lesions and 1105 mammograms of 987 women with normal and healthy breasts. We further divided our development set into testing and validation with the ratio of 8:2.

### Preprocessing

**Fig. 1:**
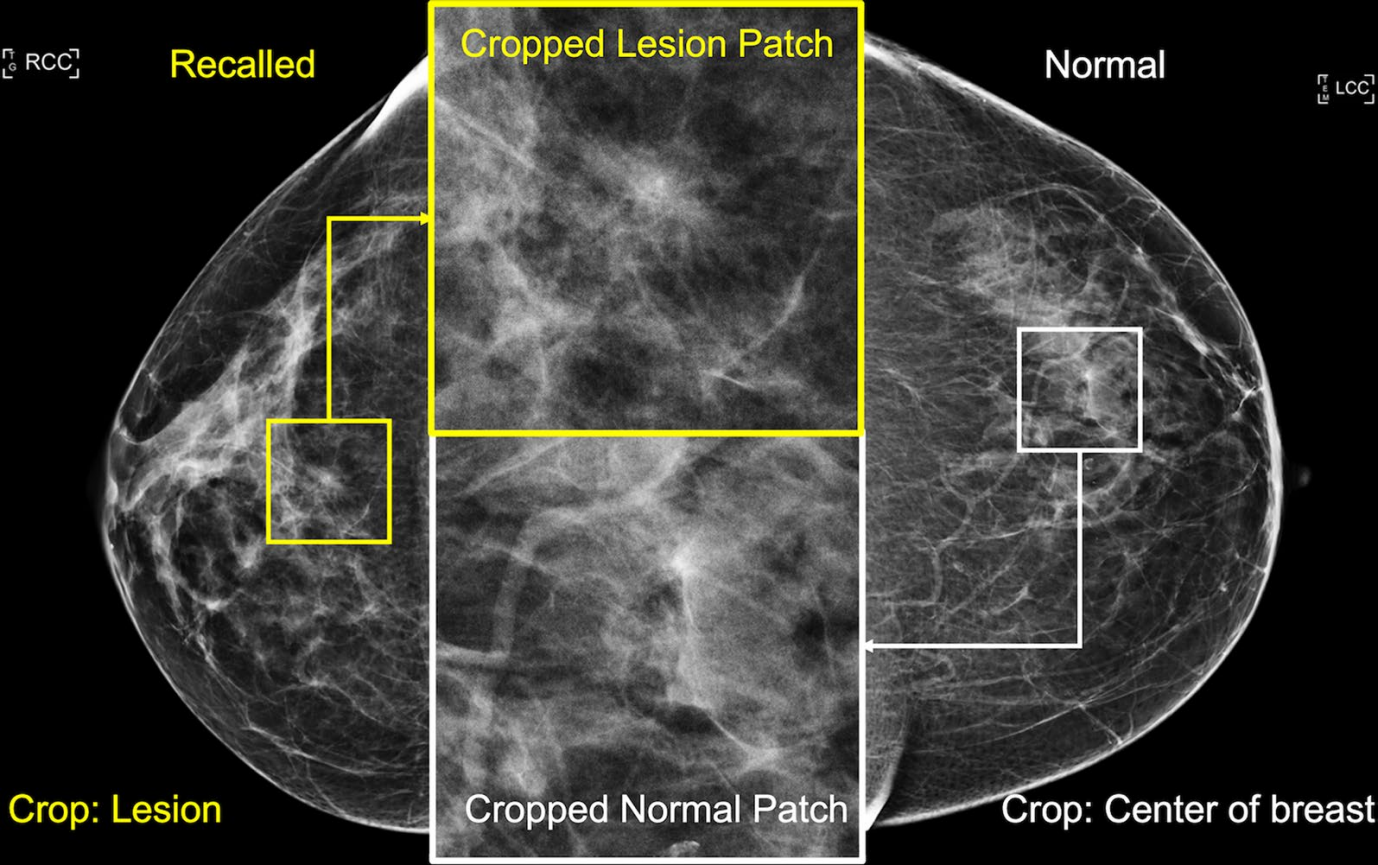
Example lesion and normal patches. This figure illustrates how we extracted 400 by 400 pixel patches from mammograms. For the cases with recalled lesions, we segmented the patch including the lesion. For normal controls, we extracted the centroid of the breast area

Using the lesion locations marked by MQSA radiologists, we segmented the patches to a size of 400 by 400 pixels (2.8 cm by 2.8 cm in size), including the recalled lesions for the cases. For normal controls, we segmented the same 400 by 400 pixel patch from the centroid of the breast area. We treated patches from the same woman, but different views (e.g., MLO and CC), as independent samples for the development dataset (training and validation). For testing, we randomly selected only one image patch from each patient to prevent possible data correlation between two different views of the same lesion. Figure 1 illustrates the above preprocessing process.

### Cycle-GAN

A Cycle-GAN consists of two generators, one for the mapping function G:X → Y and another for the mapping function F:Y → X, where X and Y are two different image domains. We set the dataset of normal patches as the source domain X, and recalled lesion patches as the target domain Y.

The loss function of the Cycle-GAN for this study is given as:

$$L\left(G, F,{D}_{X}{,D}_{Y}\right)={L}_{GAN}\left(G, {D}_{Y},X,Y\right)+{L}_{GAN}\left(F, {D}_{X},Y,X\right)$$

1 $$+{{\lambda }_{1}L}_{Cyc}\left(G,F\right)+{{\lambda }_{2}L}_{Idenity}\left(G,F\right),$$

where L_GAN_, L_Cyc_, and L_Identity_ refer to the adversarial loss, the cycle-consistency loss, and the identity loss, respectively. In addition, λ_1_ and λ_2_ are the weights that control the relative importance of L_Cyc_ and L_Identity_ compared to L_GAN_.

With associated generator *Gen*, discriminator *Dis*, and images in two domains, L_GAN_ can be formulated as follow:

2 $${L}_{GAN}\left(Gen, Dis,X,Y\right)={\mathbb{E}}_{x}\left({\text{log}}Dis\left(x\right)\right)+{\mathbb{E}}_{y}\left({\text{log}}\left(1-Dis\left(Gen\left(y\right)\right)\right)\right),$$

where *Gen* and *Dis* refer to generator and discriminator. x and y are samples from two image distributions X and Y. *Gen* and *Dis* are optimized adversarially, that is, ${min}_{Gen}{max}_{Dis}$ ${L}_{GAN}(Gen, Dis, A,B)$. In this study, we used *G−D*_*Y*_ and *F−D*_*X*_ as *Gen* and *Dis* pairs, and *X* and *Y* as images in two different distributions/domains.

L_Cyc_ was introduced to ensure the consistency of style-transferred images, i.e., images translated from X to Y, and then back again to X, should be similar to X and vice versa. L_Cyc_ can be formulated as:

3 $${L}_{Cyc}\left(G,F\right)={\mathbb{E}}_{x}\left({\Vert F\left(G\left(x\right)\right)-x\Vert }_{1}\right)+{\mathbb{E}}_{y}\left({\Vert G\left(F\left(y\right)\right)-y\Vert }_{1}\right).$$

L_Identity_ is the loss that restricts the mapping within the same domain as nearly identical when providing the real samples from one domain to the corresponding generator (i.e., G:Y → Y and F:X → X). This loss preserves the original characteristics of the real samples after the generator. L_Identity_ can be formulated as:

4 $${L}_{Identity}\left(G,F\right)={\mathbb{E}}_{x}\left({\Vert F\left(x\right)-x\Vert }_{1}\right)+{\mathbb{E}}_{y}\left({\Vert G\left(y\right)-y\Vert }_{1}\right).$$

### Lesion remover

Once the Cycle-GAN is trained, the two mapping functions G and F can transfer the style from one domain to another domain. As we used patches with normal tissue and with a recalled lesion as the images in two independent domains, the generator with mapping function *G* will work as the ***lesion simulator*** by translating the normal patch to be similar to the lesion patch. Likewise, the generator with the mapping function *F* will work as the ***lesion remover*** by changing the style of the lesion patch to that of a normal patch. We refer to generator *G* as the ***lesion simulator*** and generator F as ***the lesion remover***. Note that the focus of this paper is using the lesion remover as the lesion highlighter to improve the detection performance of CADe algorithms in mammograms. Discussing the potential use of the lesion simulator is beyond the scope of this paper.

**Fig. 2:**
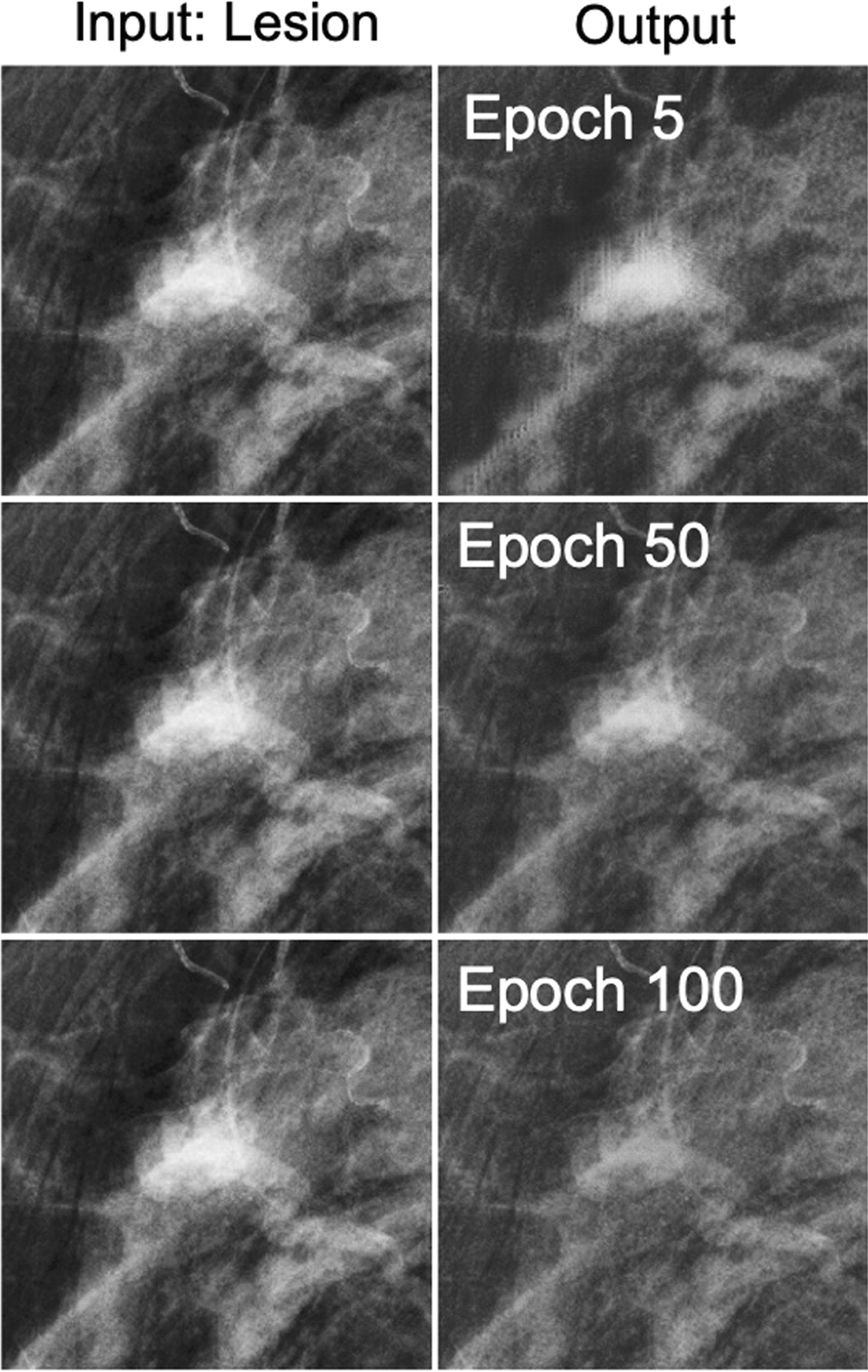
The lesion remover outcomes over the course of the training. Images in the first column show the patch with a recalled lesion for epochs 5, 50 and 100, and images in the second column are their corresponding output results. As the training epoch number increases, the lesion remover starts working as expected; the lesion remover removes or makes the existing lesion subtle

We optimized the Cycle-GAN using an Adam optimizer [14] with a learning rate of 0.0002, and momentum parameters of β_1_ = 0.5, β_2_ = 0.999. In addition, we set the maximum epoch as 100 and the weights for L1 regularization, λ_1_ and λ_2_, as 10 and 0.5, and a minibatch size of 4. We used a random left–right vertical flip as data augmentation. We used a Nvidia Titan X GPU with a 12 GB memory for training the networks. Figure 2 shows the simulation results from the lesion remover over the course of the training.

### Lesion remover as lesion highlighter

Once trained, the lesion remover can remove the existing lesion in a given mammogram. We hypothesized that one can combine an image with a lesion removed with its original to highlight the existing lesion, such that a CADe algorithm can detect the lesion better from the combined images than that from the original.

**Fig. 3:**
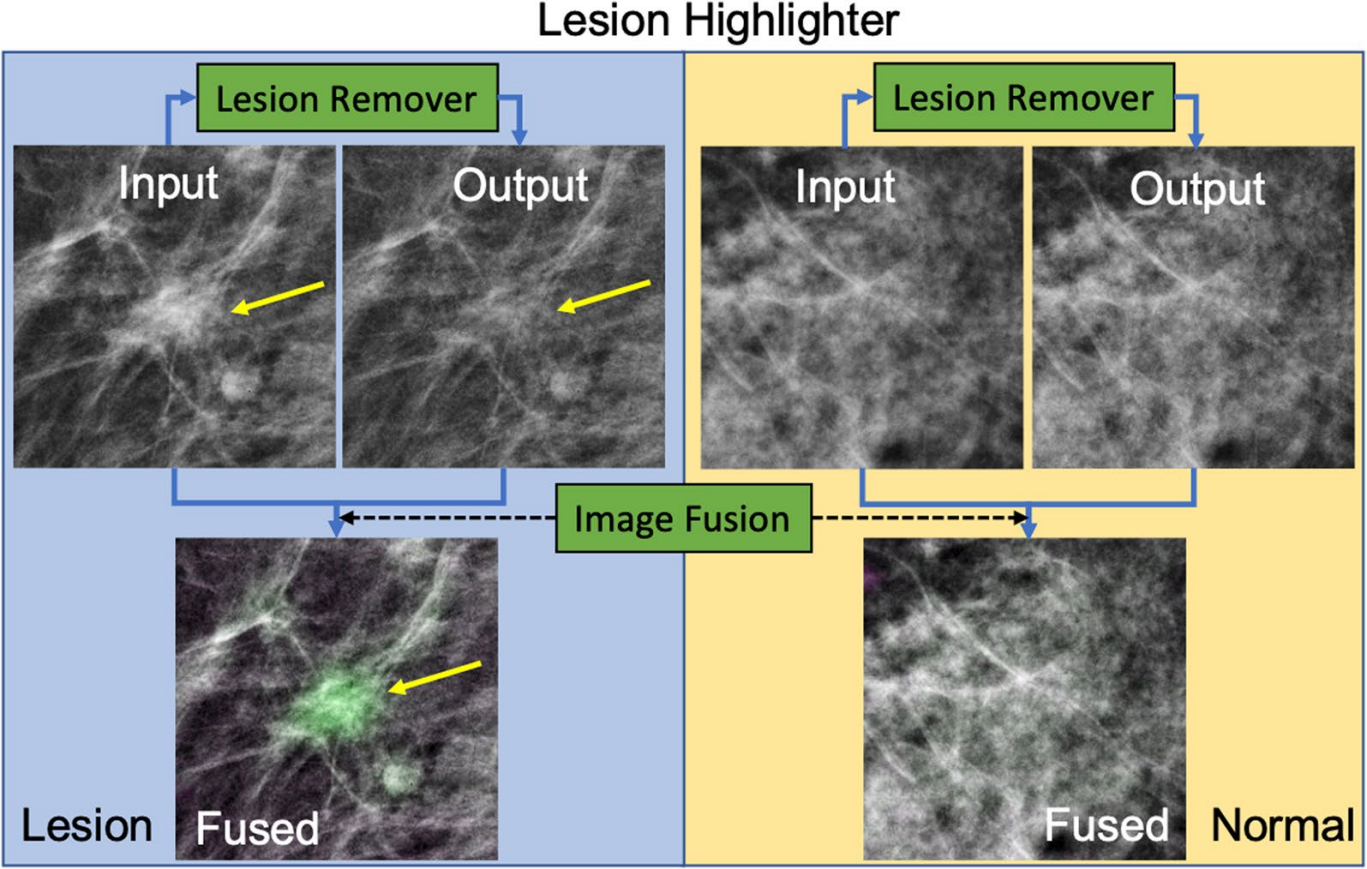
Explanation of the Lesion Highlighter. This figure illustrates how we used the lesion remover as a lesion highlighter to increase the contrast of a given lesion to its background. The left side of this figure shows when the lesion highlighter was applied to a case that contains a lesion, while the right side of the figure shows a normal control image. The yellow arrow indicates the location of a recalled lesion. After applying the lesion remover on the given input image, we fused the image with its original to create a lesion highlighted image, as shown in the bottom left. Note that the lesion remover on the normal tissue kept the original characteristics intact such that there was no highlight shown in the resulting image on the bottom right

We used the color fusion scheme (*imfuse* in MATLAB) to combine the lesion removed image with its original. The color fusion scheme we used colorizes the pixel value (green or magenta) if the image pixel values from two images were different, while retaining the gray value for those with the same pixel values. As the lesion remover should remove the lesion only, while keeping the other tissue intact, the resulting color fused image should highlight the lesion as shown in Fig. 3. Hence, the ***lesion remover*** can be used as a ***lesion highlighter*** if we combine the lesion removed with its original.

Note that one may think the lesion remover is not effective on images with normal tissue, as it was trained to remove lesion-like appearances in a mammogram, which may create a false positive detection by falsely enhancing normal tissue. However, the Cycle-GAN has an identity loss to ensure the F(x) **≈** x and G(y) **≈** y as shown in Eq. (4), such that the generator F is unlikely to remove any lesion-like normal breast tissue.

We applied the above lesion highlighter scheme on both image patches with normal and recalled lesions. Figure 3 illustrates how we applied the lesion highlighter for improving computer-aided detection of lesions.

### Lesion detector

We used various state-of-the-art deep learning architectures for image classification as our lesion detector to classify the given image patch as a recalled lesion or normal. We employed ResNet18 [15], DenseNet201 [16], EfficientNetV2 [17], and Vision Transformer (ViT) [18]. All the networks we used were pretrained on ImageNet [19].

We updated the last few layers of each ImageNet pretrained network to match our purpose; to classify the patch as a recalled lesion or not. We then used the images from the training set to train each network. We refer to these networks trained on original mammogram patches as *baseline*. Likewise, we trained each network using the training set after the lesion highlighter was applied. We refer to these networks as *highlighted*. We validated the networks after each training epoch using the validation set. As the input size of all networks was 224 by 224 pixels, we randomly segmented 224 by 224 patches from the original patch images with 400 by 400 pixels. In addition, we employed random vertical and horizontal flips, random rotation with ± 30º, and random scales with ± 25%.

For training ResNet18 and DenseNet201, we used the MATLAB training environment. Specifically, we used the Adam optimizer [14] with an initial learning rate of 0.001, a learning rate dropping factor of 0.1 for every 10 epochs, and momentum parameters of β_1_ = 0.5, β_2_ = 0.999. In addition, we set the maximum epoch as 50 and a minibatch size of 128. We also employed early stopping when the validation accuracy at each epoch dropped more than 5 times. For training EfficientNetV2 and ViT, we used the Pytorch training environment [20] with a similar augmentation setup to that of MATLAB, except for the number of epochs for ViT, which we set to 100 epochs. We used a Nvidia Titan X GPU with a 12 GB memory for training all networks.

### Evaluation methods

**Fig. 4:**
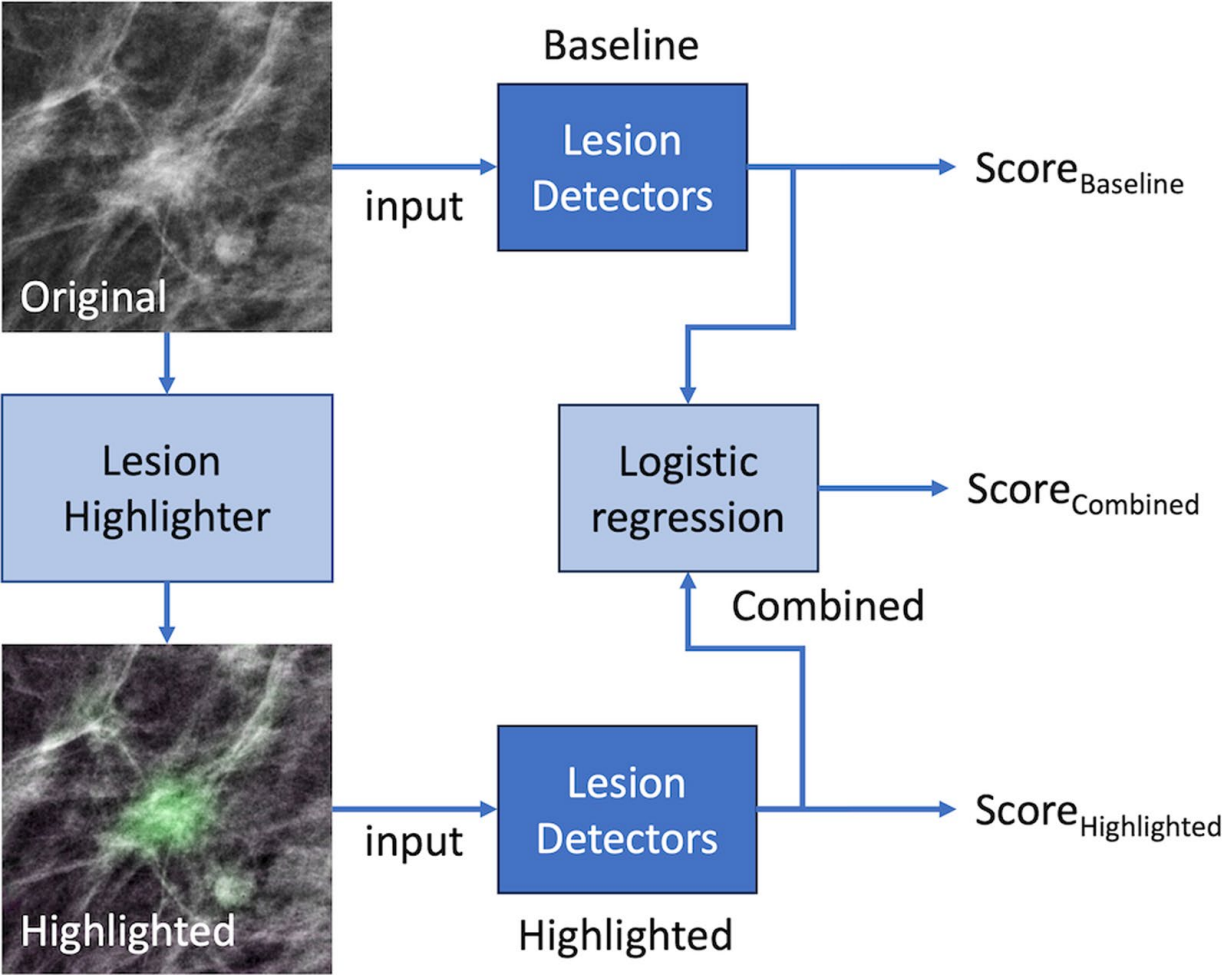
Explantion of lesion detectors. This figure illustrates how we train lesion detectors using the original and lesion highlighted lesions. We used four different deep network architectures including ResNet18, DenseNet201, EfficientNetV2, and Vision Transformer (ViT) as our lesion detector. For each detector, we built *Baseline* model using original patch, *Highlighted* model using highlighted patch, and *Combined* by combining the scores from Baseline and Highlighted using logistic regression

We refer to the network trained solely on original mammograms as *baseline* (or *base*), and those which trained on lesion highlighted mammograms as *highlighted* (or *hi-lited*). It is possible that mammograms before and after applying the lesion highlighter would provide different but complementary information for lesion detection. Therefore, we developed a logistic regression classifier to combine the diagnostic information between the baseline and the highlighted versions. Specifically, we trained the logistic regression classifier using the scores of both versions on the validation set. We then referred the resulting logistic regression classifier for each network that we considered as *combined* (or *comb*). Figure 4 illustrates how we constructed *baseline*, *highlighted*, and *Combined* lesion detectors for this study.

We used the Area under the Receiver Operating Curve (AUC) for classifying a given patch as containing a lesion or not as our figure of merit. Note that our hypothesis is that the lesion highlighter would increase the AUC of a classifier in identifying patches containing a lesion. Hence, for each CNN architecture, we compared the performances of the highlighted and combined models over the baseline model using Delong’s method [21].

## Results

### Evaluation on the effectiveness of the lesion highlighter

**Fig. 5:**
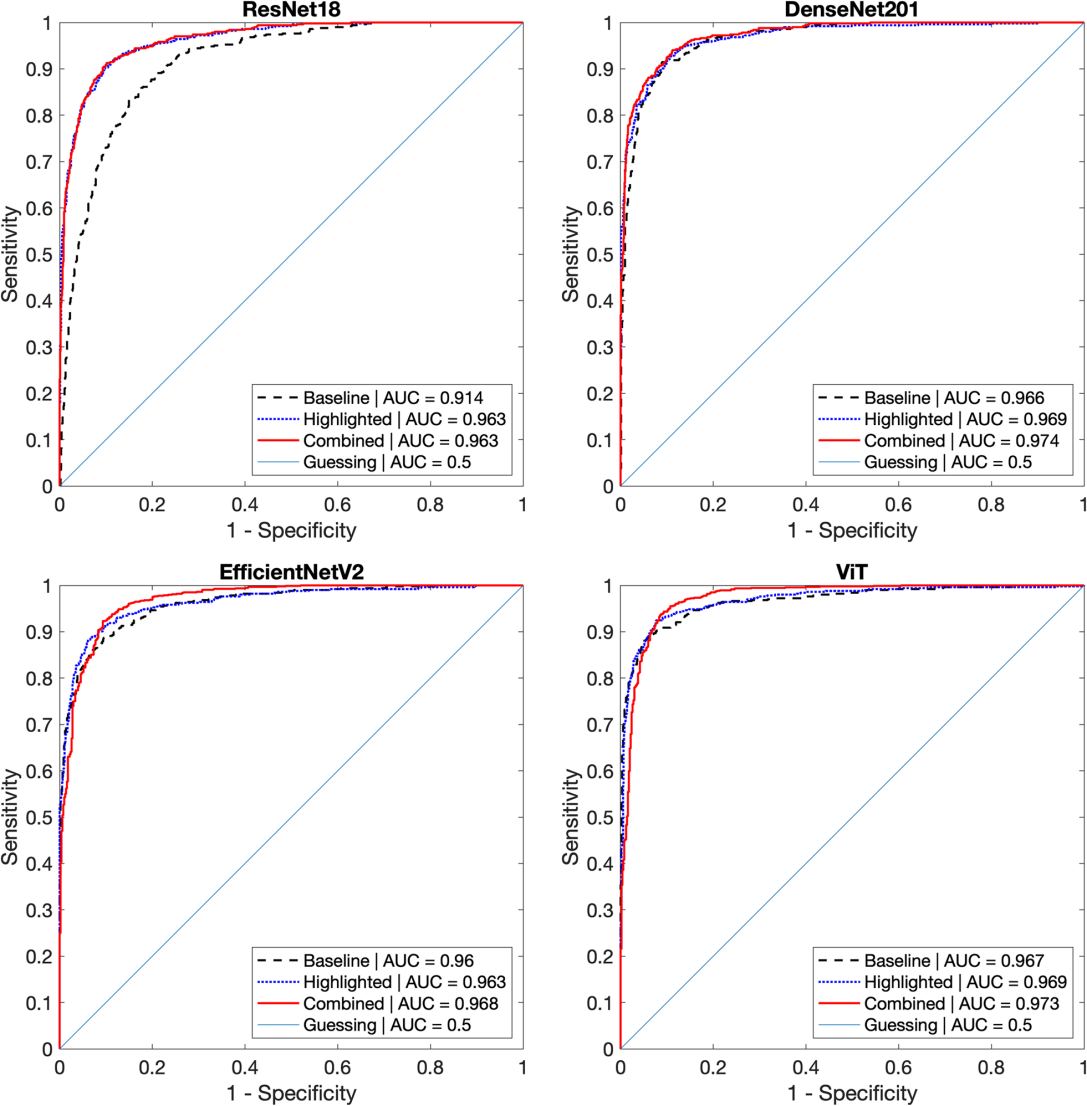
The ROC curves and associated AUCs of lesion detection networks on the test set. The test set included 507 recalled lesion and 987 normal tissue patches. Among deep network architectures considered in this study, ViT performed best over other architectures, regardless of its versions (Baseline, Highlighted, and Combined). We found the effectiveness of our proposed lesion highlighter for all architectures. Specifically, for ResNet18, both *Highlighted* and *Combined* versions performed better than its *Baseline* version (p < 0.0001, Table 1). For other more advanced and complex state-of-the-art networks, *Combined* versions performed better than their *Baselines* (p < 0.0001, Table 1)

Figure 5 and Table 1 show the ROC curves and their AUCs for *Baseline, Highlighted,* and *Combined* versions of deep networks we employed on the test set, including 504 patches with recalled lesions and 936 patches with normal breast tissue. Among all architectures and their versions, ViT performed generally best (Table 1) on the test set, although we cannot claim a statistical significance of its performance over other networks.

**Table 1:** Detection performances of various CNN architectures on non-highlighted and highlighted versions, and their differences

Model	Type	AUC on Test set		Diff. over base [95% CI]	p-value
ResNet18	Baseline	0.914			N/A
	Highlighted		0.963	0.049 [0.036, 0.062]	< 0.0001^*^
	Combined		0.963	0.050 [0.038, 0.061]	< 0.0001^*^
DenseNet201	Baseline	0.966			N/A
	Highlighted		0.969	0.003 [-0.004, 0.010]	0.426
	Combined		**0.974**	0.008 [0.004, 0.012]	< 0.001^*^
EfficientNetV2	Baseline	0.96			N/A
	Highlighted		0.963	0.003 [-0.005, 0.011]	0.476
	Combined		0.968	0.008 [0.004, 0.012]	< 0.001^*^
ViT	Baseline	**0.967**			N/A
	Highlighted		**0.969**	0.002 [-0.005, 0.009]	0.553
	Combined		0.973	0.006 [0.003, 0.010]	< 0.001^*^

^*^Statistically significant after Bonferroni correction with adjusted critical p-value of 0.006

We found the most significant performance improvement on ResNet18 by using our *lesion highlighter.* Specifically, ResNet18_Base_ (i.e., without the lesion highlighter) achieved an AUC of 0.914. After applying the lesion highlighter, the detection performance of ResNet18_Hi-lited_ improved to an AUC of 0.963. By combining the *Highlighted* and *Baseline* versions using a logistic regression, the resulting network (ResNet18_Comb_) achieved an AUC of 0.963. We found that the *Highlighted* and *Combined* versions of ResNet18 performed similarly to each other. However, both networks performed statistically better (p < 0.0001, Table 1) than its Baseline with differences in AUC of 0.049.

We found higher sensitivity for high specificity levels compared to those at lower specificity for ResNet18. Specifically, the sensitivity at a specificity of 0.98 (SE@SP98) for ResNet18 _Hi-lited_ was 0.681 and SE@SP98 for ResNet18_Comb_ was 0.677, while that of ResNet18_Base_ was only 0.345. Their differences were 0.331 and 0.329, which were statistically significant (p < 0.0001). This suggests that our *lesion highlighter* was effective on highlighting subtle breast lesions where the *baseline* ResNet18 was not able to detect the lesion.

For other state-of-the-art architectures, we found that the detection performances before and after applying the lesion highlighter were similar to each other (p-value > 0.426, Table 1). However, we found that the *highlighted* and *baseline* versions were processing different information in the mammogram such that they complemented each other for the lesion detection task. Specifically, the detection performance of the *combined* version (*highlighted* + *baseline*) with logistic regression was statistically better than that of its *baseline* (p-values < 0.001, Table 1). These results indicate that the lesion highlighter is effective regardless of the choice of network architectures, as it could provide additional information for lesion detection.

For EfficientNetV2 and ViT architectures, we found a higher improvement in the specificity value for a high sensitivity level, when their baseline and highlighted networks were combined. Specifically, the specificity value at a sensitivity of 0.98 (SP@SE98) for EfficientNetV2_Comb_ was 0.760 and the SP@SE98 of ViT_Comb_ was 0.815. However, their baseline models achieved only SP@SE98 values of 0.620 and 0.569. Their differences (Combined – Baseline) were 0.145 for EfficientNetV2 and 0.246 for ViT, which were statistically significant (p < 0.0001). These results suggest that our *lesion highlighter* could provide additional information over the original mammograms such that it helped advanced deep models to discern a difficult normal case better than before the lesion highlight.

### Indepth analysis on the effectiveness of the Lesion Highlighter for recalled lesions

**Fig. 6:**
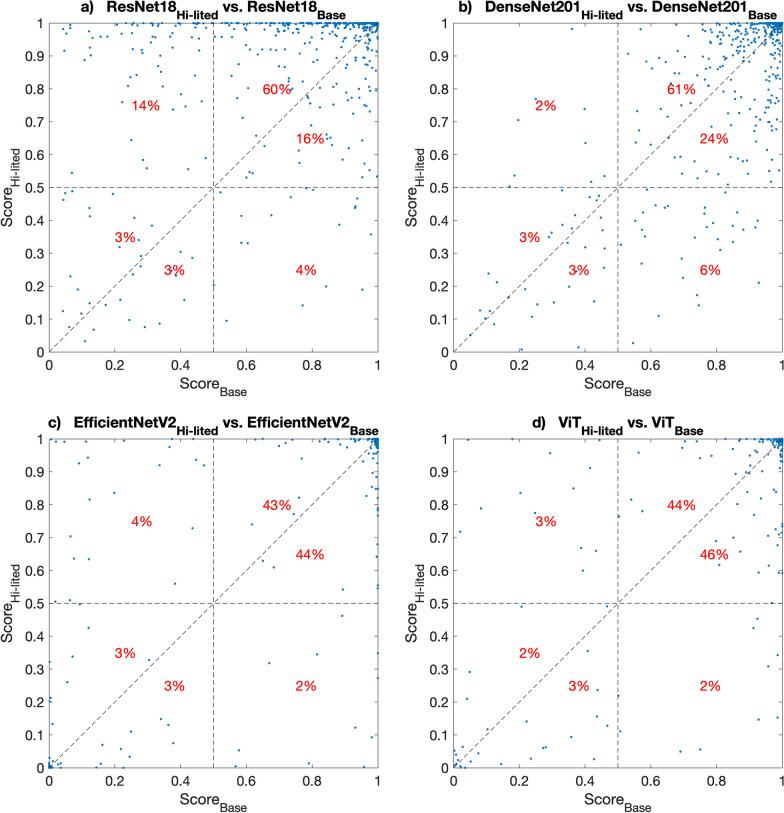
The scatter plots of Highlighted and Baseline scores on recalled lesions. This figure shows the scatter plots of scores by all models on images with recalled lesions before (x-axis) and after (y-axis) applying the lesion remover as a lesion highlighter. The points above the diagonal line indicate the cases where the lesion remover effectively highlighted the lesion such that the corresponding lesion scores were increased

To evaluate how the lesion highlighter effectively highlighted possible lesions, we conducted post-hoc analysis using the test data. To do so, we first evaluated how the detector’s lesion score changed after applying the lesion highlighter. Figure 6 shows the scatter plots of the scores of the *highlighted* and *baseline* versions of each model for the recalled lesions. For this analysis, we focused on the score differences between the *highlighted* and *baseline versions*, as we can identify in which cases the lesion highlighter is effective or provides different but additional information over its *baseline* when they (i.e., *baseline* and *highlighted*) are combined.

For ResNet18, we found that the lesion scores of 77% (60% + 14% + 3%) of the recalled lesion cases in the test set increased (i.e., improved) after applying our lesion highlighter. Scores for 16% (upper right quadrant and below diagonal line) of the lesion cases decreased after applying the lesion highlighter, but the resulting scores were still higher than 0.5, making them a correct identification of the lesion using 0.5 as the threshold. Note that many cases were concentrated at the top end, where the scores of ResNet18_Hi-lited_ were close to 1, while those of ResNet18_Base_ were clearly less than 1, indicating the effectiveness of the proposed method in highlighting the lesion location.

For other models, there were less lesion cases with significantly improved scores compared to those of ResNet18 ((a)14% vs. (b)2%, (c)4%, and (d) 3%, see upper left quadrant of each subplot in Fig. 6). In addition, the scores for most lesion cases were concentrated in the upper right quadrant (80% or higher), indicating both versions correctly identified most lesion cases using 0.5 as the threshold. These results partially confirm the results on the ROC curves and AUC values in Fig. 6. That is, unlike ResNet18 with the most significant lesion score improvement (14%, upper left quadrant in Fig. 6a), the performances of the *highlighted* and *baseline* versions of the other models were similar to each other for identifying recalled lesions (see the area of high specificity area, i.e., the left portion of ROC curves in Fig. 6c–d. As a result, there was less improvement in classifying more recalled lesions correctly when its *combined* version was applied on those cases.

**Fig. 7:**
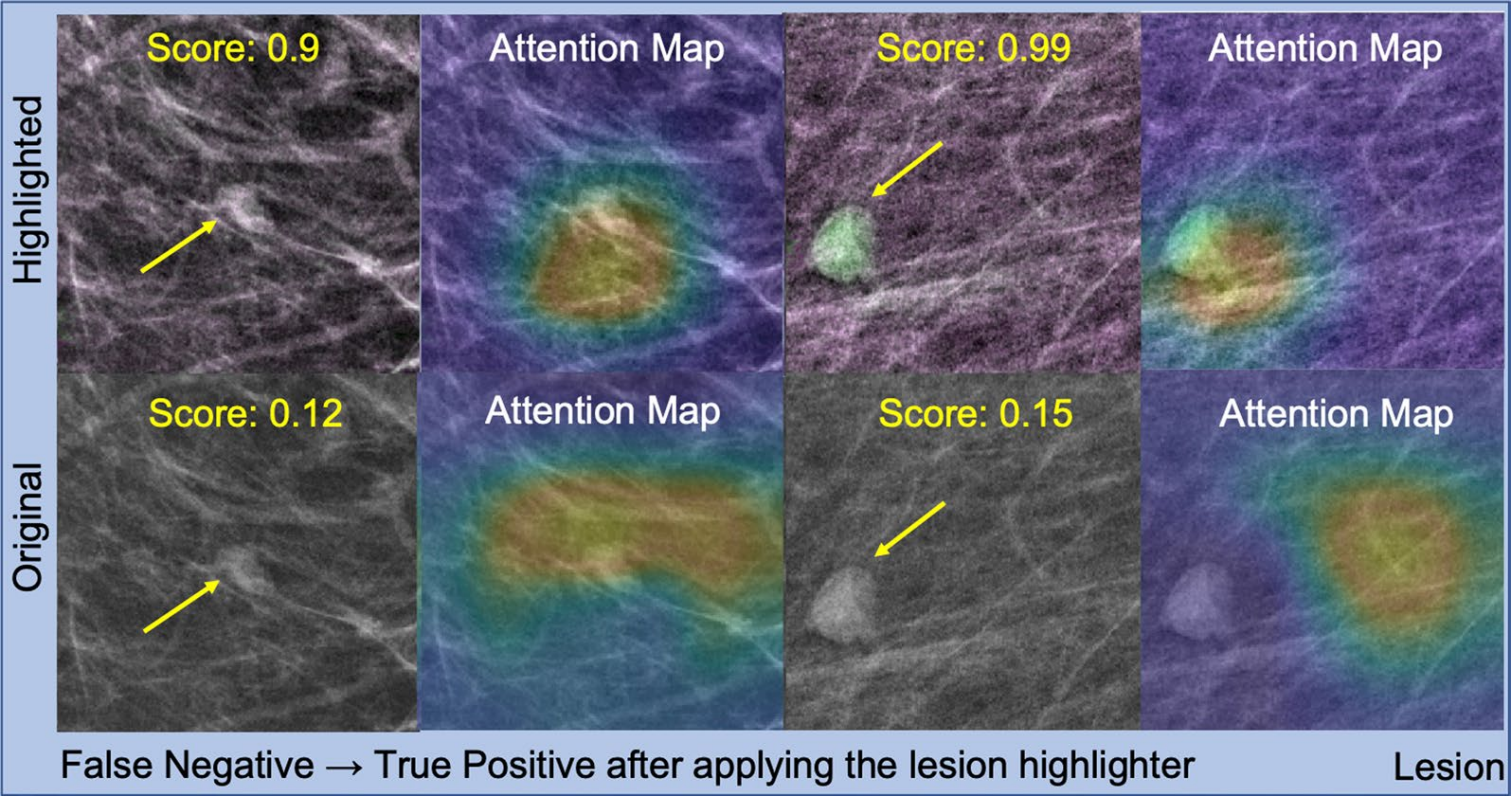
False negative lesion cases that changed to true positive after applying the lesion highlighter. This figure shows two lesion cases that were false negative before applying the lesion highlighter but changed to true positive after applying the lesion highlighter. The first and third column show the images with recalled lesions after (top row) and before (bottom row) applying the lesion highlighter. The second and last column show the attention map by the detector. The yellow arrows indicate the location of the recalled lesions. Before applying the lesion highlighter, the detector was not able to localize the lesion but after the lesion highlight, it correctly localized the lesion

We then visually inspected a few cases where the lesion highlighter effectively highlighted lesions that were previously missed by the lesion detector. For this visual inspection, we used ResNet18 as the representative model, as the results of other models were similar. Figure 7 shows two cases in the higher right quadrant in Fig. 6, which were false negative detections by ResNet18_Base_ on non-highlighted images (i.e., original images) but became true positives by ResNet18_Hi-lited_ after the lesion highlighted. The images in the first and third columns show the input image for lesion detectors and the images in the second and fourth columns are the attention map (using Grad-CAM [22]) of each lesion detector. We found that the cases that initially were false negative before applying our lesion highlighter were too subtle to be detected. However, our lesion highlighter effectively highlighted them by increasing their contrast to the background by increasing pixel intensity (first column) or applying different colors (third column) such that the detector was able to locate the lesion correctly with a high lesion score.

### Indepth analysis on the effectiveness of the Lesion Highlighter for normal controls

**Fig. 8:**
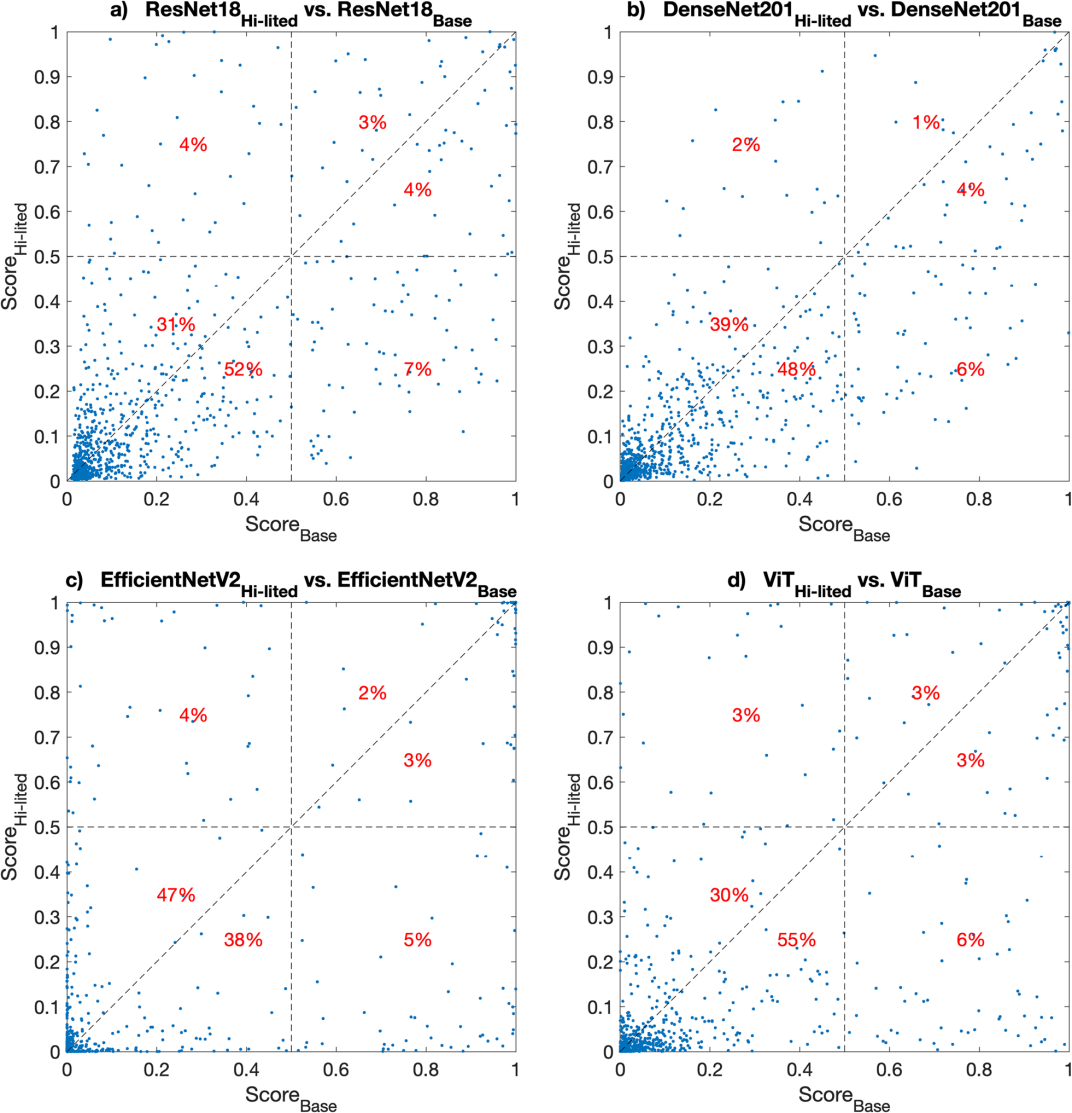
The scatter plot of Highlighted and Baseline scores on normal controls. This figure shows the scatter plot of scores by all models on the normal control images before (x-axis) and after (y-axis) applying the lesion remover as a lesion highlighter. The points below the diagonal line indicate the cases where the lesion highlighter effectively changed the appearance of lesion-like normal tissue such that the corresponding lesion scores decreased

We repeated the post-hoc analysis as the above on normal controls. Figure 8 shows the scatter plots of the scores by the *highlighted* and *baseline* versions of all considered models on the normal controls in the test set. The points below the diagonal line are the cases that the proposed lesion highlight was effective.

For ResNet18, we found that our lesion highlight method was effective for a total of 63% (52% + 7% + 4%) of normal controls (Fig. 8a). Specifically, 7% of normal controls (lower right quadrant) that were falsely identified as positive cases (false positive) before were correctly classified as normal controls after applying the lesion highlighter. Most normal controls (31%) where the proposed method was less effective were located in the lower left quadrant, especially near the origin. This indicates that, although the lesion highlighter increased the lesion score of the normal controls, such negative impact is minimal as they are still lower than the traditional lesion threshold of 0.5.

For DenseNet201 and ViT models, we found a similar trend for the case of ResNet18; the scores for 58% and 64% of the normal controls decreased (i.e., improved) after the lesion highlighter, respectively. For EfficientNetV2, although there was a smaller number of improved cases for the normal controls (47%), all scores were concentrated at the origin, showing the similar performance of both versions. In addition, 5% or higher of normal controls (lower right quadrant in Fig. 8b–d) that were falsely identified as positive cases (false positive) before were correctly classified as normal controls after the lesion highlighter, which was less than the number of opposite cases (2% vs. 6% for DenseNet201, 4% vs. 5% for EfficientNetV2, and 3% vs 6% for ViT, Fig. 8b–d).

**Fig. 9:**
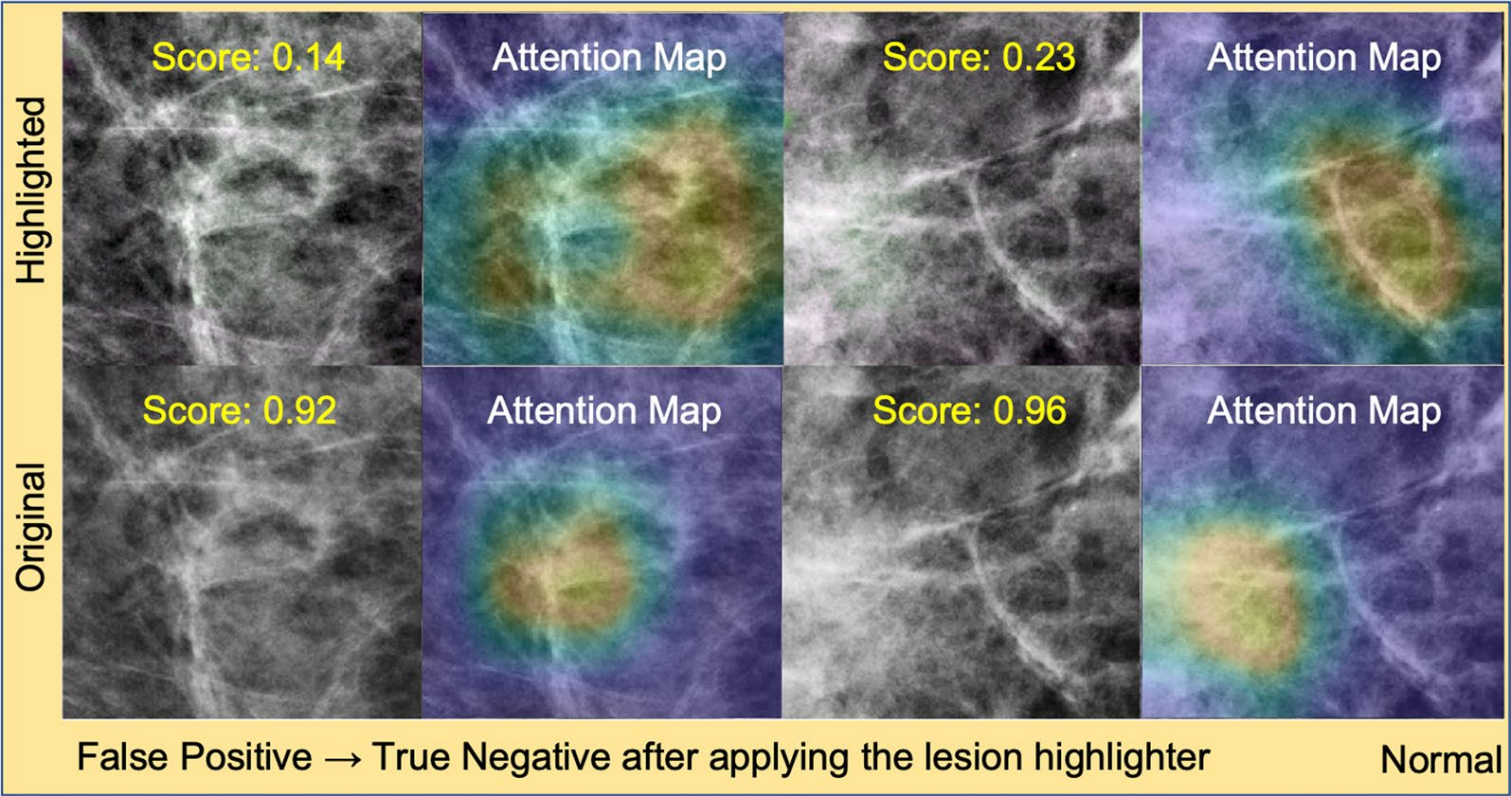
False positive normal controls that changed to true negative after applying the lesion highlighter. This figure shows two normal controls which were false positive before applying the lesion highlighter but changed to true negative after applying the lesion highlighter. The first and third column show the images with normal tissue with (top row) and without (bottom row) applying the lesion remover. The second and last column show the attention map by the detector. Before applying the lesion highlighter, the detector was falsely focused on the normal tissue as a lesion, but after the lesion highlighter, the detector’s attention was moved away from the previously falsely focused area. Note that the normal breast tissue after applying the lesion highlighter show pixels in green lightly but widely spread in the image, which made the detector correctly identify as normal tissue

Like the recalled lesion cases, we visually inspected a few normal controls where our lesion highlighter was effective on the samples that had previous false positive findings. Similarly, we used ResNet18 as a representative model for this visual inspection. Specifically, Fig. 9 shows two cases in the lower right quadrant in Fig. 8, which were false positive detections by ResNet18_Base_ on non-highlighted images (i.e., original images) but became true negative by ResNet18_Hi-lited_ after applying the lesion highlighter. The images in the first and third column show the input image for lesion detectors and the images in the second and fourth columns are the attention map of each lesion detector. We found that the detector was falsely focused on normal tissue as lesions before applying the lesion highlighter. But after applying the lesion highlighter, the detector’s attention was moved away from the areas where it was falsely focused for incorrect decisions (i.e., false positive detection). We found that wide areas of breast tissue were highlighted lightly in green, which made the detector correctly identify them as normal tissue.

Based on the above post-hoc analysis (Figs. 6, 7, 8, 9), when it is effective, our lesion highlighter successfully located recalled lesions and highlighted their location without falsely highlighting the location of lesion-like normal tissue; instead, it helped the detector to make correct decisions on previously false positive findings.

## Discussion

In this study, we developed a Cycle-GAN based ***lesion remover*** by training it on image patches with recalled lesions and normal breast tissue. We showed that the ***lesion remover*** can be used as a ***lesion highlighter*** by contrasting the resulting images to their originals. Specifically, the lesion remover removed existing lesions, such that we highlighted the existing lesion location in mammograms by color-fusing the lesion removed image with its original. To show the effectiveness of the ***lesion remover*** as a ***lesion highlighter***, we developed four lesion patch detectors using state-of-the-art deep network architectures, including ResNet18, DenseNet201, EfficientNetV2, and ViT, one trained on images after the lesion highlighter was applied (*highlighted*), another without the lesion remover (*baseline*), and those two combined (*combined*) by training a logistic regression classifier on top of two networks. We found that the *combined* version of all considered networks achieved statistically better detection performance than their *baseline* versions, which were trained on original mammograms without the lesion highlighter applied.

It is important to note that our lesion highlighter is computationally effective. One can find that the most significant improvement was shown for ResNet18 (Fig. 5 and Table 1). Its baseline performance was lowest compared to other networks. However, with the lesion highlighter applied, its performance (AUC of ResNet18_Hi-lited_ was 0.963) was comparable to those of more complex and deeper network architectures, such as ViT (AUC of ViT_Hi-lited_ was 0.969). Considering the amount of computational power consumed to optimize those advanced networks, our lesion highlighter is effective, as it made the simple and low computational cost network show comparable performance to those of state-of-the-art architectures.

However, there were occasions when our proposed method was less effective, or even failed. Specifically, there were 5% to 9% of recalled lesion cases (lower left and right quadrants and below the diagonal line of each subplot in Fig. 6) where our approach was less effective. However, half of them (lower left quadrant but below diagonal line) were difficult cases to detect, as both the *highlighted* and *baseline* versions estimated them as non-lesions. The remaining half indicates the cases when the lesion highlighter failed; the true positive detection was incorrectly changed to false negative after applying the lesion highlighter.

**Fig. 10:**
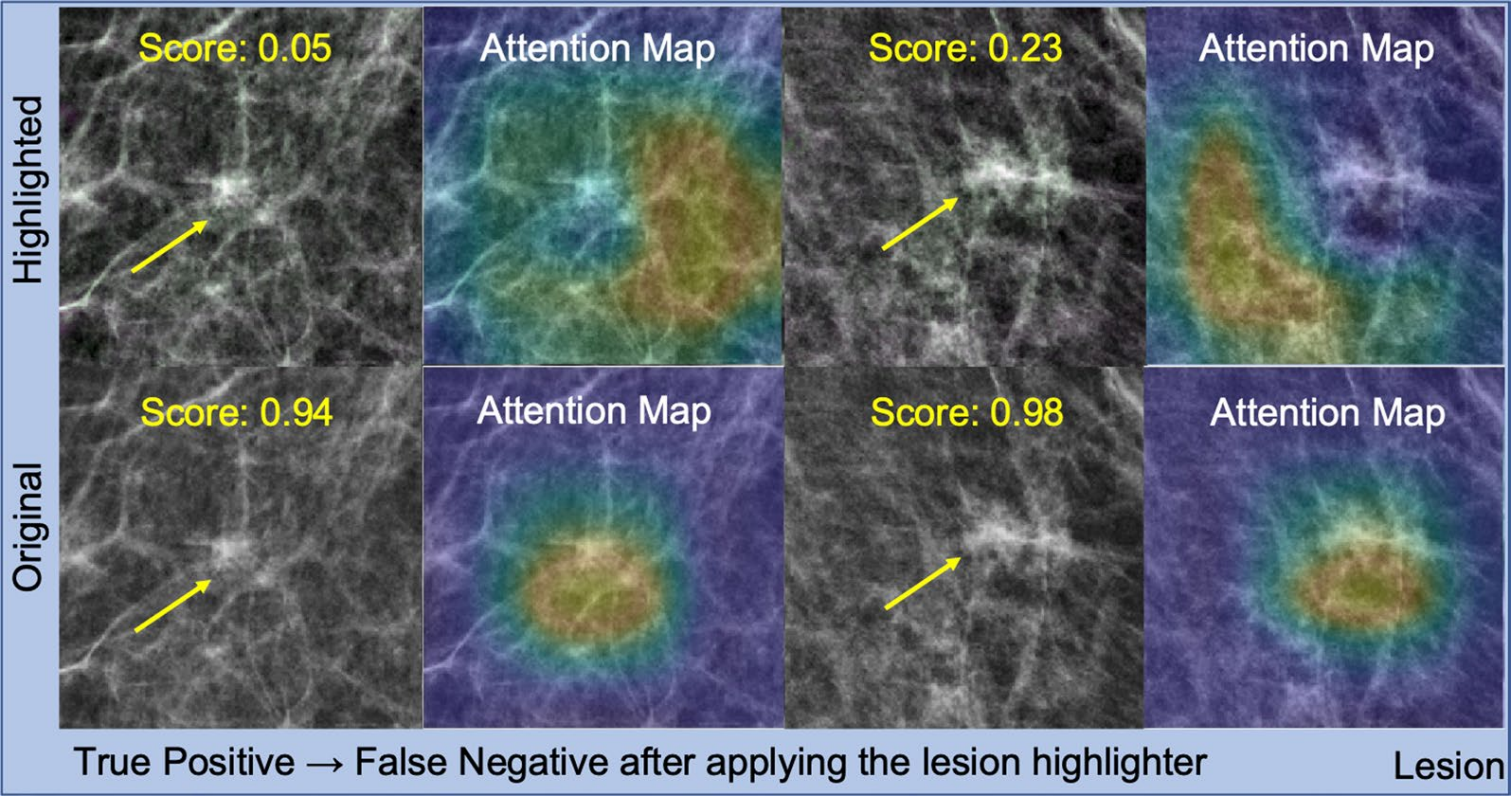
True positive lesion cases that changed to false negative after applying the lesion highlighter. This figure shows two lesion cases which were true positive detection before applying the lesion highlighter but changed to false negative after applying the lesion highlighter. The first and third column show the images with recalled lesions with (top row) and without (bottom row) applying the lesion highlighter. The second and last column show the attention map by the detector. We found that our method failed to locate the lesion such that it falsely highlighted a wider area of the breast tissue in green. As a result, the lesion detector failed to recognize the lesion from the image

We then sampled and visually inspected two representative lesion cases among those that failed in Fig. 10. Like previous visual inspections, we used ResNet18 for this analysis. We found that the lesion remover failed to localize the lesions (which were located at the center of the image) and, therefore, it removed normal tissue more than it was supposed to do. As a result, a wider area of breast tissue than the lesion was highlighted (in green), which moved the correct focus of the lesion detector on the lesion (attention maps in the bottom row, Fig. 10) away from its true location (attention maps in the top row, Fig. 10).

**Fig. 11:**
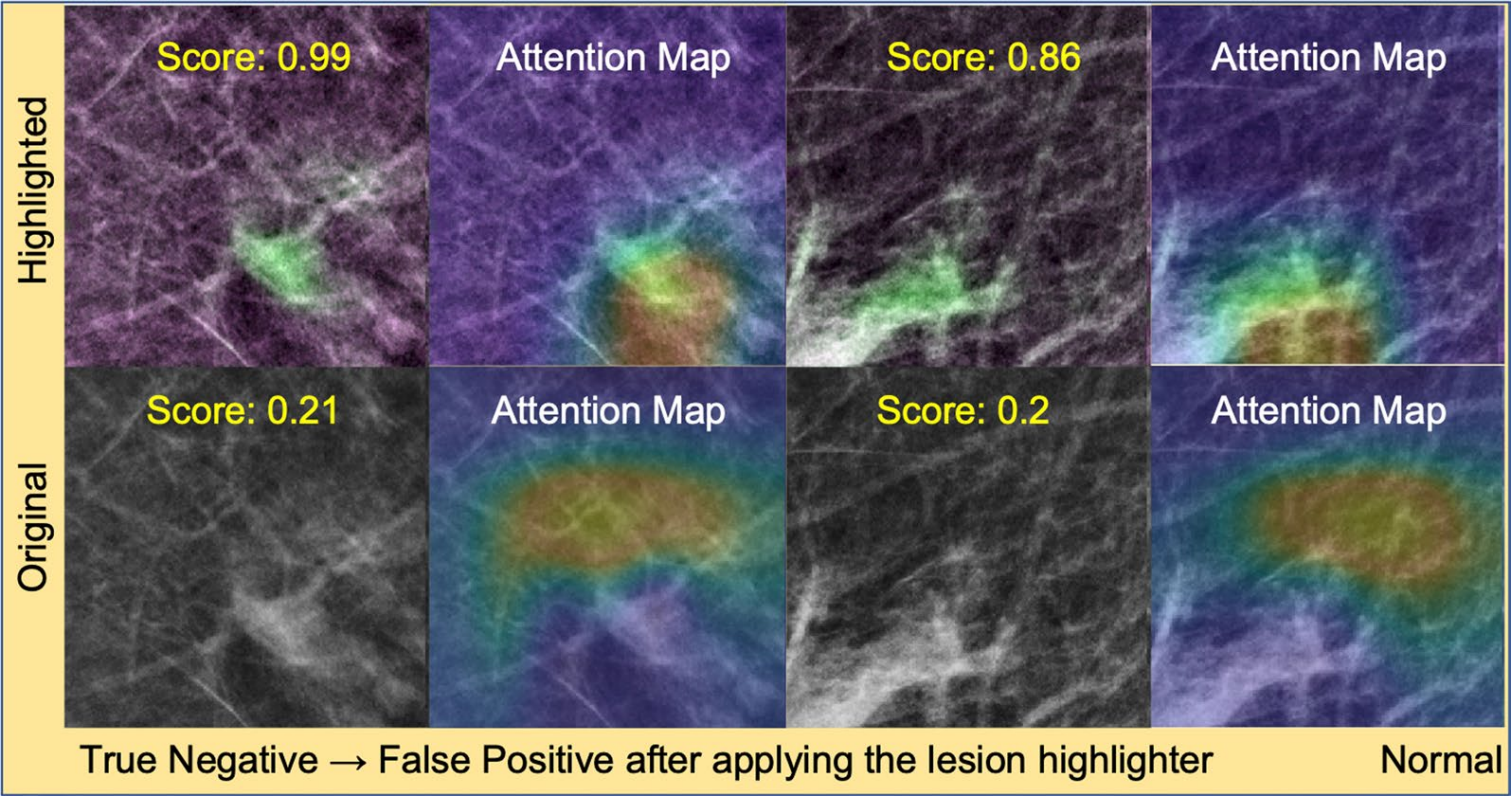
True negative normal controls that changed to false positive after applying the lesion highlighter. This figure shows two normal controls which were true negative before applying the lesion highlighter but changed to false positive after applying the lesion remover. The first and third column show the images with normal tissue with (top row) and without (bottom row) applying the lesion highlighter. The second and last column show the attention map by the detector. We found that our method falsely identified the normal breast tissue as lesions such that it was falsely highlighted in green, which made the lesion detector identify it as a lesion

We found that our lesion highlighter was less effective for only 1% to 4% of normal controls (upper left quadrant of each subplot in Fig. 8). Like the above lesion cases, we inspected two representative normal controls where the lesion remover failed (Fig. 11). We found that the lesion remover falsely identified normal breast tissue as a lesion such that it was incorrectly highlighted in green.

The reason for the above failed cases was due to an error made by the lesion remover on the given images, i.e., false negative and false positive detections, resulting in the false highlighting of normal tissue. Specifically, the detector made false negative predictions when lesions and normal background tissue were highlighted together (Fig. 10). For normal controls, lesion like normal tissue was falsely highlighted (Fig. 11). These false highlights of normal tissue could be a limitation of our approach.

A possible reason for the false highlighting of normal tissue could be due to the limited number of samples we used to train our lesion remover. Although we used over 10 k samples to train the model, it could not cover the characteristics of all possible lesions and normal tissues. Specifically, we segmented the center of the breast area to prepare image datasets for normal controls. We can include other breast areas with dense tissue for training. For this, one can utilize breast density segmentation algorithms (e.g., [23–25]) to identify challenging breast dense tissue for our purpose. Having more recalled lesions and normal breast tissue samples will improve our lesion remover, such that it could reduce the above false highlighting of normal tissue. We will investigate this in a future study.

In addition, the ground truth (or labeling) of the positive samples was noisy, as we used recalled lesions, which included lesions with different malignancy levels (straight benign, biopsied benign, malignant) and different lesion types (masses, calcifications, architectural distortions, etc.). As we mentioned earlier, we only knew the lesion truth (BI-RADS 0 or 1) at the time of screening. We will investigate the lesion details from radiology and pathology reports in the future. Having such lesion details will allow us to develop various lesion removers specialized for each lesion malignancy level and type. For example, we can develop a malignancy mass (or calcs) remover to develop a malignancy mass (or calcs) highlighter to help CADx algorithms.

There is room for improvement in our method. Specifically, we developed a lesion remover using image patches with the size of 400 by 400 pixels (2.8 cm × 2.8 cm). It is big enough to include various types of lesions, but it is still patch based and therefore, additional work is required to scale our findings to the level of a full mammogram. There are two possible ways to realize lesion removers (as well as lesion highlighters) in full mammograms. First, we can directly apply our method on full mammograms via windowing, but within the breast area only, as our lesion remover may not work on the breast boundary (close to breast skin), since our current lesion remover was never trained on such areas. Second, we can directly develop a lesion remover directly from full size mammograms, like the work of Zhou et al. [8]. In fact, our previous study already showed that simulating high resolution mammograms using GAN is possible [26]. Thus, we will develop a lesion remover (and supsequently lesion highlighter) for full mammograms by investigating the above options.

## Conclusions

We developed a lesion remover using a Cycle-GAN trained on image patches from recalled lesions and normal breast tissue. We showed that the lesion remover can be operated as a lesion highlighter if we contrast the images after the lesion is removed with their original. For shallow networks, like the ResNet18 detector, a lesion highlighter can help the detector by finding more lesions that were previously missed while reducing false positive detections. For more advanced architectures, like the Vision Transformer detector, a lesion highlighter can help the detector by discerning difficult normal cases that were previously identified as lesions. In addition, a lesion highlighter is computationally effective as it improves the performance of a shallow ResNet18 to the level of a state-of-the-art architecture.

## Data Availability

All data needed to evaluate the conclusions in the paper are present in the paper. The datasets (such as original screening mammography images and their related BI-RADS assessments) used and/or analyzed during the current study are available from the corresponding author on reasonable request. A signed data use agreement and institutional review board approval will be required before the release of research data.

## Abbreviations

LH: Lesion Highlighter
LR: Leison Remover
ROC: Receiver Operating Characteristic
GAN: Generative Adversarial Network
MQSA: Mammography Quality Standards Act
I2I: Image-to-Image
CADe: Computer-aided detection
CADx: Computer-aided diagnosis
OCR: Optical Character Recognition
MRI: Magnetic Resonance Imaging
AI: Artificial Intelligence
BI-RADS: Breast Imaging-Reporting and Data System
SE@SP98: Sensitivity at a specificity of 0.98
SP@SE98: Specificity at a sensitivity of 0.98
